# Levels of Salivary IFN-gamma, TNF-Alfa, and TNF Receptor-2 As Prognostic Markers in (Erosive) Oral Lichen Planus

**DOI:** 10.1155/2010/847632

**Published:** 2011-02-13

**Authors:** Noha A. Ghallab, Naglaa El-Wakeel, Olfat G. Shaker

**Affiliations:** ^1^Department of Oral Medicine, Periodontology and Diagnosis, Faculty of Oral and Dental Medicine, Cairo University, 43 Zahraa Street, Dokki, Giza 12311, Egypt; ^2^Department of Oral Medicine and Periodontology, Faculty of Dental Medicine, Al Azhar University, Cairo, Egypt; ^3^Department of Medical Biochemistry, Faculty of Medicine, Cairo University, Egypt

## Abstract

To explore the feasibility of detecting salivary levels of IFN-*γ*, TNF-*α*, and sTNFR-2 from erosive oral lichen planus (ELP) patients for clinical application, 20 ELP patients were enrolled in the study as were 20 age-sex-matched controls. From all subjects, saliva level of the tested biomarkers was determined by ELISA. Salivary profiles were assessed in ELP patients by ELISA after being treated with prednisone. A significantly higher level of IFN-*γ* (*P* ≤ .01), TNF-*α* (*P* ≤ .0001), and sTNFR-2 (*P* ≤ .01) was detected in ELP patients before treatment than in controls. Following treatment, the salivary levels of IFN-*γ* (*P* ≤ .01), TNF-*α* (*P* ≤ .05), and sTNFR-2 (*P* ≤ .01) decreased significantly when compared to their pretreatment levels. This study demonstrated that salivary IFN-*γ*, TNF-*α*, and sTNFR-2 can be detectable in ELP patients and decreased significantly after treatment with prednisone, which may reveal the possibility of using these disease-related biomarkers in diagnosis and monitoring.

## 1. Introduction

Oral lichen planus (OLP) is a chronic inflammatory condition involving the oral mucosal tissues [1]. Although the mechanisms of OLP pathogenesis have not been fully disclosed, it has been suggested that autoreactive cytotoxic CD8^+^ T-cells trigger keratinocytes apoptosis in OLP [2]. Evidence has shown that a complex cytokine network plays an important role in the exacerbation and perpetuation of OLP; hence, TNF-*α* and IFN-*γ* have been extensively studied and were proven by many investigators to have a key regulatory role in the immunopathogenesis of OLP [3, 4]. Being a Th1 cytokine, IFN-*γ* is involved in the activation of CD8^+^ cytotoxic T-cells and maintains keratinocytes major histocompatibility class II expression in OLP, thereby contributing to disease chronicity [2, 5]. 

Cell-mediated immunity in OLP may be regulated by various cytokines and their receptors [5]. TNF-*α* is a highly pleiotropic, multifunctional cytokine that regulates diverse cellular responses via binding to two distinct cell surface receptors: TNF receptor-1 (TNFR-1/60 kDa) and TNF receptor-2 (TNFR-2/80 kDa) [6]. Apoptosis is seen as a general TNF-*α*-mediated cytotoxic phenomenon occurring through the TNFR-1 signaling pathway [7]. Sugerman et al. [2] suggested that one of the possible mechanisms used by CD8^+^ cytotoxic T-cells to trigger keratinocyte apoptosis in OLP included the T-cell-secreted TNF-*α* binding to TNFR-1 on the keratinocyte surface. This binding may activate the keratinocyte caspase cascade resulting in keratinocyte apoptosis. 

Previous studies showed that many cell types coexpress TNFR-1 and TNFR-2 and require collaboration between these two receptors to generate TNF-*α*-mediated apoptosis [8, 9]. Furthermore, several investigations have reported that TNFR-2 may play an important role in the induction of TNFR-1-mediated apoptosis [10, 11]. Accordingly, it would be of interest to study the soluble TNFR-2 (sTNFR-2) levels in OLP patients, given that to the best of our knowledge, sTNFR-2 profiles have only been assessed in the serum of patients with cutaneous LP [12].

Saliva has more potential diagnostic value that is generally appreciated and has promise to be an aid in the diagnosis of systemic and oral diseases in the last decade [13]. Whole saliva offers distinctive advantages over other research mediums such as serum, lesional tissues, and lesional transudates in that individuals with modest training could repeatedly collect it noninvasively [14]. Hence, it provides a cost-effective approach for disease monitoring and screening of large populations [15]. Whole saliva has been successfully applied for detection of proinflammatory cytokines and adhesion molecules in several immunologically mediated diseases [16, 17]. 

It is important to evaluate the practical application of salivary biomarkers' analyses in clinical management of OLP [18]. Accordingly, several studies in the literature have demonstrated the significance of OLP-related cytokines in monitoring treatment [4, 19]. However, minimal studies have explored the potential of cytokines' receptors in OLP patients for clinical application. Consequently, the purpose of the current study was to investigate the possibility of detecting different disease-related biomarkers' including TNF-*α*, IFN-*γ*, and sTNFR-2 in saliva of erosive OLP (ELP) patients and to monitor the therapeutic response by determining their levels before and after treatment with a systemic corticosteroid, prednisone.

## 2. Material and Methods

### 2.1. Study Population

The entire study sample comprised 40 subjects: 20 patients suffering from ELP (18 female, 2 male; range 40 to 55 years) and 20 healthy controls (17 female, 3 male; range 41 to 56 years). The patients were diagnosed with ELP; while the controls were healthy normal individuals free form any systemic disease or inflammatory oral lesions. The distribution of subjects according to the age and gender is presented in Table 1.

### 2.2. Inclusion and Exclusion Criteria

Subjects were selected from the Outpatient Clinic, Department of Oral Medicine and Periodontology, Faculty of Oral and Dental Medicine, Cairo University. A detailed medical history of each subject was obtained according to the detailed questionnaire of the modified Cornell Medical Index [20]. All subjects were free from any systemic disease and did not receive any medication either topical or systemic that could cause lichenoid reaction during the 3 months before the study. Moreover, patients with suspected restoration-related reaction or gingival inflammation were excluded from this study. Written consent was obtained from each subject who signed an informed consent form approved by the University Institutional Review Board. This was followed by an explanation of the study as well as information about the treatment and follow-up appointments needed.

All patients had oral symptoms, and every case was clinically diagnosed as ELP. Duration of the disease ranged from 2 to 3 months with periods of remission and exacerbation. The erosive lesions were bilateral and extended to involve the buccal mucosa, labial mucosa, and tongue which varied from one patient to the other. Diagnosis was confirmed by histopathologic examination according to the World Health Organization's (WHO's) clinicopathological diagnostic criteria for LP [21]. 

According to Carbone et al. [22], ELP patients were treated with systemic prednisone, at 40–60 mg/day in a single morning dose for variable lengths of time, anyhow not up to 60 days. When an almost 50% reduction in lesion size was achieved, the prednisone dose was tapered by reducing 10 mg each week and finally to 5 mg/day for the last week. Patients were checked every week, and the dose and length of the treatment were adjusted in each case following the clinical needs. Patients were instructed not to use any other medication for the treatment of ELP during this study period.

### 2.3. Collection of Samples

#### 2.3.1. Salivary Sample Collection

Collection of whole unstimulated saliva (WUS) was done using standard techniques according to Navazesh [23]. At the time of saliva collection, lesions were actively symptomatic. Salivary samples were obtained in the morning and subjects were asked not to eat, brush their teeth, or use mouth rinse at least 2 hours prior to salivary sample collection on that day. Samples were obtained by requesting subjects to swallow first, tilt their head forward, and expectorate 10 mL of unstimulated whole saliva into a sterile centrifuge tube. After collection, the saliva was immediately centrifuged for 2 min at 10,000 × g and the clarified supernatant was filtered through a 0.45 *μ*m low protein binding membrane, separated into 0.5 mL aliquots and frozen at −80°C until assayed.

#### 2.3.2. Detection of Salivary Levels of IFN-*γ*, TNF-*α*, and sTNFR-2

The evaluation in this study included assessing the levels of IFN-*γ*, TNF-*α*, and sTNFR-2 in saliva of patients with ELP before and after treatment with prednisone. After collecting all the salivary samples, the studied biomarkers were assessed by ELISA.

#### 2.3.3. Determination of IFN-*γ* in Salivary Samples

Saliva samples were determined by using a commercially available enzyme-linked immunosorbent assay (ELISA) kit (R&D Systems Inc., Minneapolis, MN, USA). This assay employs the quantitative sandwich enzyme immunoassay technique. A polyclonal antibody specific for IFN-*γ* has been precoated onto a microplate. Standards and samples (100 *μ*L each) were pipetted into the wells in duplicate, and any IFN-*γ* present was bound by the immobilized antibody. After washing away any unbound substances, an enzyme-linked polyclonal antibody specific for IFN-*γ* (200 *μ*L) was added to the wells for 2 hours. Following a wash to remove any unbound antibody-enzyme reagent, a substrate solution (200 *μ*L) was added to the wells for 30 min and color developed in proportion to the amount of IFN-*γ* bound in the initial step. The yellow color development was stopped, and the intensity of the blue color was measured by measuring absorbance at 450 nm, with the correction wavelength set at 540 nm or 570 nm. The minimum detectable dose (MDD) of IFN-*γ* was typically less than 8.0 pg/mL. The concentrations of samples were calculated from the standard curve, and the results were presented in picogram per milliliter (pg/mL). The concentrations of the curve were ranged from 0–1000 pg/mL.

#### 2.3.4. Determination of TNF-*α* and sTNFR-2 in Saliva

Levels of TNF-*α* and sTNFR-2 in saliva samples were determined by using a commercially available enzyme-linked immunosorbent assay (ELISA) kit (R&D Systems Inc.). These are standard “sandwich” ELISAs and were performed using human recombinant standards according to the manufacturer's instructions. These assays employ the quantitative sandwich enzyme immunoassay technique. A monoclonal antibody specific for TNF-*α* and sTNFR-2 has been precoated onto a microplate. Standards and samples (100 *μ*L each) in duplicate, were pipetted into the specific wells, and any TNF-*α* and/or sTNFR-2 present was bound by the immobilized antibody. After washing away any unbound substances, an enzyme-linked polyclonal antibody (200 *μ*L) specific for TNF-*α* and sTNFR-2 was added to the specific wells. Following a wash to remove any unbound antibody-enzyme reagent, a substrate solution (200 *μ*L) was added to the specific wells and yellow color developed in proportion to the amount of TNF-*α* and/or sTNFR-2 bound in the initial step. The color development was stopped, and the intensity of the blue color was measured. These assays have a lower limit of detection for TNF-*α* (1.6 pg/mL) and for sTNFR-2 (0.6 pg/mL). Concentrations of the cytokine and its soluble receptor in the sample were determined by generation of a standard curve for comparison, and results were presented in pg/mL. The TNF-*α* standard curve ranged from (0–1000 pg/mL) while that of sTNFR-2 ranged from (0–500 pg/mL)

### 2.4. Statistical Analysis

Results were expressed as mean ± standard deviation (SD) and median. First “one way ANOVA” was performed to demonstrate the difference of each biomarker level between ELP and control group in saliva. Then, “repeated measures ANOVA” was used for comparison of the change of each biomarker level in saliva before and after treatment in ELP group. All data were processed with a computerized statistical package (SPSS 15.0 for Windows, SPSS Inc., Chicago, IL.) (MedCalc Software 11.4.4 for Windows, Mariakerke, Belgium).

## 3. Results

**Figure 1:**
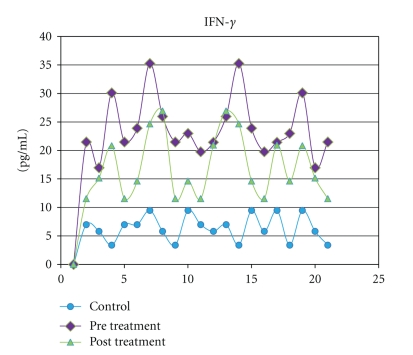
Salivary levels of IFN-*γ* measured in pg/mL from control subjects compared with levels in ELP patients before and after treatment with prednisone.

**Figure 2:**
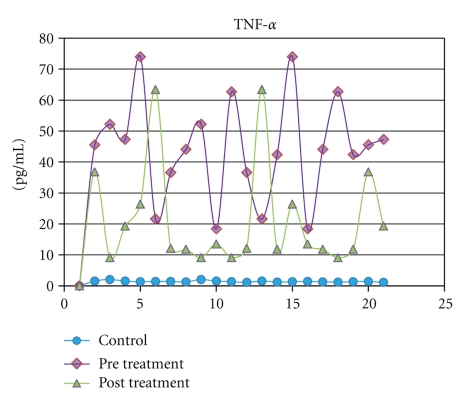
Salivary levels of TNF-*α* measured in pg/mL from control subjects compared with levels in ELP patients before and after treatment with prednisone.

**Figure 3:**
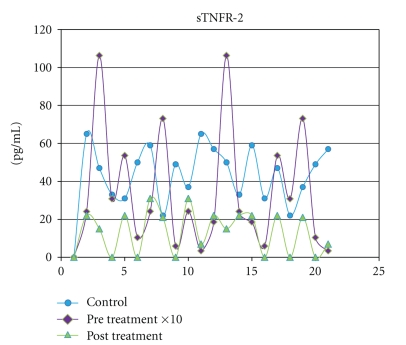
Salivary levels of sTNFR-2 measured in pg/mL from control subjects compared with levels in ELP patients before and after treatment with prednisone.

IFN-*γ*, TNF-*α*, and sTNFR-2 were detected in all saliva samples obtained from healthy subjects. The levels of the IFN-*γ*, TNF-*α*, and sTNFR-2 (*P* = .009) in saliva increased remarkably in the ELP group than those in the controls. The highest significance (*P* = .0001) was observed when both TNF-*α* and IFN-*γ* concentrations in saliva were compared to their corresponding control groups (Table 2). Following treatment with systemic prednisone, the salivary levels of IFN-*γ* (*P* = .001), TNF-*α* (*P* = .0263), and sTNFR-2 (*P* = .01) decreased significantly when compared to their pretreatment levels (Table 2 and Figures 1, 2, and 3).

## 4. Discussion

Salivary assays have shown to have a significant correlation with lesional tissue partners [24] and serum partners in OLP patients [14]. Considering that whole saliva is a complex mixture derived from the salivary glands, along with contributions from the gingival crevicular fluid (GCF) and other sources [13], and that biochemical markers can be secreted through GCF, therefore, subjects in this study were selected without any gingival inflammation that could confound the present results using saliva as the research media. As the enhanced expression of disease-related cytokines and their soluble receptors has been demonstrated by previous reports to contribute to the pathogenesis of OLP [4, 5, 16], it is reasonable for clinicians to focus on the clinical application of detecting these biomarkers. Accordingly, the current study focused on the salivary profiles of INF-*γ*, TNF-*α*, and sTNFR-2 in ELP patients in response to treatment with prednisone in an attempt to reveal the possibility of their use in disease diagnosis and monitoring. Since systemic corticosteroids are indicated for patients with severe erosive or atrophic OLP [22] and wide spread lesions [25] owing to their anti-inflammatory and immunosuppressive effects [26, 27], prednisone was chosen in this study being the most widely used systemic corticosteroid in treatment of OLP with high rates of efficacy (80–100%) [22]. 

The present study demonstrated a statistically significant elevation in salivary levels of TNF-*α* and INF-*γ* in OLP samples compared to normal control. These data were consistent with their enhanced expression previously reported from different media in OLP patients which supports their important role in the pathogenesis of OLP [3, 5, 28, 29]. Our findings were also supported by other studies investigating the salivary levels of TNF-*α* in OLP patients [14, 16, 18]. Moreover, Pezelj-Ribaric et al. [30] demonstrated significantly higher amounts of salivary TNF-*α* in OLP patients compared to healthy controls which even showed correlation with disease severity being significantly higher in the erosive/atrophic type than in reticular type of OLP. The NF-*κ*B-dependent cytokines TNF-*α*, IL-1, IL-6, and IL-8 were also detected at significant elevated levels in three types of oral fluids, including saliva, from OLP patients [16]. Zhang et al. [14] even suggested that salivary assay may be a more sensitive method to reflect the enhanced disease-related cytokine production when compared to serum. 

To date, the results on IFN-*γ* in OLP have been inconsistent. The data presented in this study was supported by a recent investigation showing that erythematous/ulcerated OLP patients had a significant higher level of IFN-*γ* in lesions and saliva than control [24]. On the other hand, Liu et al. [15] recently demonstrated low expression of IFN-*γ* in saliva of patients with OLP claiming that it may result from the counter-regulation of pathologically elevated Th2-derived cytokines such as IL-4, involved in cellular immunosuppression. In contrast, Khan et al. [5] could not detect in vitro IL-4 secretion in any OLP lesional T-cell lines; therefore, this suggestion still needs further research. 

The increase of salivary cytokines in ELP patients observed in this study might be due to local production from cells of the inflammatory infiltration and/or by the keratinocytes themselves [14]. It has been also suggested that T-cell activation and cytokine production act locally and are not reflected in peripheral blood [31]. Moreover, the epithelia from OLP lesions have been previously shown to produce TNF-*α* 10- to 20-fold greater than those in keratinocytes from normal gingiva [32]. 

The current study illustrated a significant decrease in the salivary levels of TNF-*α* and IFN-*γ* after treatment with prednisone which advocates the fact that they play an important role in the inflammatory process and immunopathogenesis of OLP. Accordingly, it has been reported that the number of TNF-*α*-positive mononuclear cells in erosive or atrophic OLP patients significantly decreased after treatment with 0.1% fluocinolone acetonide [4]. Recently, Youngnak-Piboonratanakit et al. [19] also demonstrated that in situ expression of IFN-*γ* was significantly elevated in OLP patients and decreased after treatment with 0.1% fluocinolone acetonide. Our results are also consistent with Rhodus et al. [18] who found statistically significant reduction in the salivary levels of NF-*κ*B-dependent cytokines in patients with ELP following treatment with dexamethasone mouth wash. The authors indicated that detection of disease-related cytokines in saliva indeed has some clinical potential in monitoring therapeutic response and disease activity status of OLP, which is strongly supported by the data presented in this study.

Considering that TNF-*α* has a role in carcinogenesis and that ELP has a greater rate of malignant evolution, this stresses on the diagnostic and prognostic potential of salivary TNF-*α* and its receptors in OLP patients [30, 33]. Our analysis, to the best of our knowledge, demonstrates for the first time that salivary levels of sTNFR-2 were significantly elevated in patients with ELP than those in healthy controls and significantly decreased after treatment with prednisone. 

TNF-*α* may trigger apoptosis in OLP through inducing differentiation and activation of CD8^+^ cytotoxic T-cells [2]. Smyth and Johnstone [34] offered an alternative view that TNF-*α* actively drives lymphoproliferation by signaling through TNFR-2. This conclusion is supported by earlier studies suggesting that TNFR-2 transmits signals important for the proliferation of cytotoxic T-cells and excluded an absolute requirement of a coexpression of TNFR-1 for TNFR-2 signaling [35, 36]. While it is clear that TNF-*α*-induced apoptosis requires TNFR-1 signaling [6], yet, numerous studies have described synergistic action of TNFR-2 and TNFR-1 in the potentiation of cell death [37–40]. It has been reported that overexpression of TNFR-2 can endow cells with the ability to undergo TNF-*α*-induced apoptosis. Several mechanisms have been proposed to explain this observation, including TNFR-2 serving as high affinity trap for TNF-*α* that delivers TNF-*α* to TNFR-1 “ligand-passing” [41], TNFR-2 assisting TNFR-1-mediated apoptosis indirectly by the induction of endogenous TNF-*α* [11, 42], and TNFR-2 enhancing and amplifying the apoptotic cell death signal transduction from TNFR-1 by promoting a caspase activating signal [43]. 

In light of the above-mentioned data, this study suggests that TNF-*α* might trigger keratinocyte apoptosis in OLP via both TNFR-1 and TNFR-2. The present study, in which the authors duly recognize that the sample size was relatively small, presents some preliminary results related to the significant expression of salivary sTNFR-2 in ELP patients which may give some new insights to the pathogenesis of OLP. Although further conclusions from these data must be interpreted cautiously, additional studies are recommended for evaluating the TNFR-1/TNFR-2 ratio and better understanding of the role of sTNFR-2 in the pathogenesis of OLP.

## 5. Conclusion

The current study demonstrated that the absolute concentrations of salivary IFN-*γ*, TNF-*α*, and sTNFR-2 can be detectable in ELP patients and decreased significantly after treatment with prednisone. The results of this study from saliva of ELP patients provide a potential for specimens to be obtained easily and noninvasively, as well as disease-related biomarkers which may reveal the possibility of their use in diagnosis and monitoring.

##  Conflict of Interests

The authors report no conflict of interests related to this study.

## Figures and Tables

**Table 1:** The characteristics of subjects.

	Age (years)		Gender
	Mean ± SD	range	F/M

OLP (*n* = 20)	46.3 ± 4.99	40–55	18/2
Control (*n* = 20)	42 ± 7.2	39–55	17/3

**Table 2:** The change of IFN-*γ*, TNF-*α*, and TNFR-2 salivary levels in ELP patients treated with prednisone and control subjects.

		IFN-*γ*	TNF-*α*	TNFR-2
ELP before treatment	Mean ± SD	23.953 ± 5.33	44.485 ± 16.81	350.4 ± 330.89
	Median	18.30	55.60	1029

ELP after treatment	Mean ± SD	17.237 ± 5.68	21.33 ± 17.21	14.0 ± 11.39
	Median	15.39	54.30	31

Control	Mean ± SD	6.41 ± 2.53	1.405 ± 0.25	45 ± 13.82
	Median	6.09	0.90	43

Before versus after	*P* value	*P* ≤ .001	*P* ≤ .05	*P* ≤ .01

Before versus control	*P* value	*P* ≤ .0001	*P* ≤ .0001	*P* ≤ .01

## References

[1] Ismail SB, Kumar SK, Zain RB (2007). Oral lichen planus and lichenoid reactions: etiopathogenesis, diagnosis, management and malignant transformation. *Journal of Oral Science*.

[2] Sugerman PB, Savage NW, Walsh LJ (2002). The pathogenesis of oral lichen planus. *Critical Reviews in Oral Biology and Medicine*.

[3] Sklavounou-Andrikopoulou A, Chrysomali E, Iakovou M, Garinis GA, Karameris A (2004). Elevated serum levels of the apoptosis related molecules TNF-*α*, Fas/Apo-1 and Bcl-2 in oral lichen planus. *Journal of Oral Pathology and Medicine*.

[4] Thongprasom K, Dhanuthai K, Sarideechaigul W, Chaiyarit P, Chaimusig M (2006). Expression of TNF-*α* in oral lichen planus treated with fluocinolone acetonide 0.1%. *Journal of Oral Pathology and Medicine*.

[5] Khan A, Farah CS, Savage NW, Walsh LJ, Harbrow DJ, Sugerman PB (2003). Th1 cytokines in oral lichen planus. *Journal of Oral Pathology and Medicine*.

[6] Tracey KJ, Cerami A (1994). Tumor necrosis factor: a pleiotropic cytokine and therapeutic target. *Annual Review of Medicine*.

[7] Idriss HT, Naismith JH (2000). TNF*α* and the TNF receptor superfamily: structure-function relationship(s). *Microscopy Research and Technique*.

[8] Weiss T, Grell M, Hessabi B (1997). Enhancement of TNF receptor p60-mediated cytotoxicity by TNF receptor p80: requirement of the TNF receptor-associated factor-2 binding site. *Journal of Immunology*.

[9] Lucas R, Garcia I, Donati YRA (1998). Both TNF receptors are required for direct TNF-mediated cytotoxicity in microvascular endothelial cells. *European Journal of Immunology*.

[10] Hess S, Engelmann H (1996). A novel function of CD40: induction of cell death in transformed cells. *Journal of Experimental Medicine*.

[11] Grell M, Zimmermann G, Gottfried E (1999). Induction of cell death by tumour necrosis factor (TNF) receptor 2, CD40 and CD30: a role for TNF-R1 activation by endogenous membrane-anchored TNF. *The EMBO Journal*.

[12] Miklòs S, Gruschwitz MS (1997). In situ expression and serum levels of tumour necrosis factor alpha receptors in patients with lichen planus. *Acta Dermato-Venereologica*.

[13] Kaufman E, Lamster IB (2002). The diagnostic applications of saliva—a review. *Critical Reviews in Oral Biology and Medicine*.

[14] Zhang Y, Lin M, Zhang S (2008). NF-*κ*B-dependent cytokines in saliva and serum from patients with oral lichen planus: a study in an ethnic Chinese population. *Cytokine*.

[15] Liu W, Dan H, Wang Z (2009). IFN-gamma and IL-4 in saliva of patients with oral lichen planus: a study in an ethnic chinese population. *Inflammation*.

[16] Rhodus NL, Cheng B, Myers S, Bowles W, Ho VU, Ondrey F (2005). A comparison of the pro-inflammatory, NF-*κ*B-dependent cytokines: TNF-alpha, IL-1-alpha, IL-6, and IL-8 in different oral fluids from oral lichen planus patients. *Clinical Immunology*.

[17] Ghallab N, Shaker O (2010). Salivary-soluble CD44 levels in smokers and non-smokers with chronic periodontitis: a pilot study. *Journal of Periodontology*.

[18] Rhodus NL, Cheng B, Bowles W, Myers S, Miller L, Ondrey F (2006). Proinflammatory cytokine levels in saliva before and after treatment of (erosive) oral lichen planus with dexamethasone. *Oral Diseases*.

[19] Youngnak-Piboonratanakit P, Dhanuthai K, Thongprasom K (2009). Expression of ifn-*γ* before and after treatment of oral lichen planus with 0.1% fluocinolone acetonide in orabase. *Journal of Oral Pathology and Medicine*.

[20] Abramson JH (1966). The cornell medical index as an epidemiological tool. *American Journal of Public Health and the Nation's Health*.

[21] Rad M, Hashemipoor MA, Mojtahedi A (2009). Correlation between clinical and histopathologic diagnoses of oral lichen planus based on modified WHO diagnostic criteria. *Oral Surgery, Oral Medicine, Oral Pathology, Oral Radiology and Endodontology*.

[22] Carbone M, Goss E, Carrozzo M (2003). Systemic and topical corticosteroid treatment of oral lichen planus: a comparative study with long-term follow-up. *Journal of Oral Pathology and Medicine*.

[23] Navazesh M (1993). Methods for collecting saliva. *Annals of the New York Academy of Sciences*.

[24] Tao XA, Li CY, Rhodus NL, Xia J, Yang XP, Cheng B (2008). Simultaneous detection of IFN-gamma and IL-4 in lesional tissues and whole unstimulated saliva from patients with oral lichen planus. *Journal of Oral Pathology and Medicine*.

[25] Al-Hashimi I, Schifter M, Lockhart PB (2007). Oral lichen planus and oral lichenoid lesions: diagnostic and therapeutic considerations. *Oral Surgery, Oral Medicine, Oral Pathology, Oral Radiology and Endodontology*.

[26] González-Moles MA, Scully C (2005). Vesiculo-erosive oral mucosal disease—management with topical corticosteroids: (1) fundamental principles and specific agents available. *Journal of Dental Research*.

[27] Lozada-Nur F, Miranda C (1997). Oral lichen planus: topical and systemic therapy. *Seminars in Cutaneous Medicine and Surgery*.

[28] Fayyazi A, Schweyer S, Soruri A (1999). T lymphocytes and altered keratinocytes express interferon-*γ* and interleukin 6 in lichen planus. *Archives of Dermatological Research*.

[29] Karagouni EE, Dotsika EN, Sklavounou A (1994). Alteration in peripheral blood mononuclear cell function and serum cytokines in oral lichen planus. *Journal of Oral Pathology and Medicine*.

[30] Pezelj-Ribaric S, Prso IB, Abram M, Glazar I, Brumini G, Simunovic-Soskic M (2004). Salivary levels of tumor necrosis factor-*α* in oral lichen planus. *Mediators of Inflammation*.

[31] Simark Mattsson C, Jontell M, Bergenholtz G, Heyden M, Dahlgren UI (1998). Distribution of interferon-*γ* mRNA-positive cells in oral lichen planus lesions. *Journal of Oral Pathology and Medicine*.

[32] Yamamoto T, Osaki T, Yoneda K, Ueta E (1994). Cytokine production by keratinocytes and mononuclear infiltrates in oral lichen planus. *Journal of Oral Pathology and Medicine*.

[33] Rhodus NL, Cheng B, Myers S, Miller L, Ho V, Ondrey F (2005). The feasibility of monitoring NF-*κ*B associated cytokines: TNF-*α*, IL-1*α*, IL-6, and IL-8 in whole saliva for the malignant transformation of oral lichen planus. *Molecular Carcinogenesis*.

[34] Smyth MJ, Johnstone RW (2000). Role of TNF in lymphocyte-mediated cytotoxicity. *Microscopy Research and Technique*.

[35] Tartaglia LA, Weber RF, Figari IS, Reynolds C, Palladino MA, Goeddel DV (1991). The two different receptors for tumor necrosis factor mediate distinct cellular responses. *Proceedings of the National Academy of Sciences of the United States of America*.

[36] Grell M, Becke FM, Wajant H, Männel DN, Scheurich P (1998). TNF receptor type 2 mediates thymocyte proliferation independently of TNF receptor type 1. *European Journal of Immunology*.

[37] Vandenabeele P, Declercq W, Vanhaesebroeck B, Grooten J, Fiers W (1995). Both TNF receptors are required for TNF-mediated induction of apoptosis in PC60 cells. *Journal of Immunology*.

[38] Declercq W, Denecker G, Fiers W, Vandenabeele P (1998). Cooperation of both TNF receptors in inducing apoptosis: involvement of the TNF receptor-associated factor binding domain of the TNF receptor 75. *Journal of Immunology*.

[39] Haridas V, Darnay BG, Natarajan K, Heller R, Aggarwal BB (1998). Overexpression of the p80 TNF receptor leads to TNF-dependent apoptosis, nuclear factor-*κ*B activation, and c-Jun kinase activation. *Journal of Immunology*.

[40] Weiss T, Grell M, Siemienski K (1998). TNFR80-dependent enhancement of TNFR60-induced cell death is mediated by TNFR-associated factor 2 and is specific for TNFR60. *Journal of Immunology*.

[41] Tartaglia LA, Pennica D, Goeddel DV (1993). Ligand passing: the 75-kDa tumor necrosis factor (TNF) receptor recruits TNF for signaling by the 55-kDa TNF receptor. *Journal of Biological Chemistry*.

[42] Vercammen D, Vandenabeele P, Declercq W, van de Craen M, Grooten J, Fiers W (1995). Cytotoxicity in L929 murine fibrosarcoma cells after triggering of transfected human p75 tumour necrosis factor (TNF) receptor is mediated by endogenous murine TNF. *Cytokine*.

[43] Chan FKM, Lenardo MJ (2000). A crucial role for p80 TNF-R2 in amplifying p60 TNF-R1 apoptosis signals in T lymphocytes. *European Journal of Immunology*.

